# Chronological age and vascular age staring at each other on the ring of cardiovascular prevention

**DOI:** 10.1016/j.ijchy.2021.100076

**Published:** 2021-01-06

**Authors:** Giacomo Pucci, Paolo Verdecchia

**Affiliations:** Department of Medicine and Surgery, University of Perugia, Perugia, Italy; Unit of Internal Medicine, Terni University Hospital, Terni, Italy; Fondazione Umbra Cuore e Ipertensione-ONLUS and Department of Cardiology, Hospital S. Maria Della Misericordia, Perugia, Italy

**Keywords:** Atherosclerosis, Pulse wave velocity, Hypertension

Ageing is a physiological process which is phenotypically characterized by the senescence of organs and tissues. Beyond appearing tautological, the sentence that “ageing is the major risk factor of age-related diseases” uncovers an important truth: that the multi-systemic expression of ageing significantly contributes to multi-morbidity [1]. This clearly applies to the cardiovascular (CV) disease. With ageing, several physiological processes which characterize the efficiency of the CV system may undergo important changes, losing part of their efficiency and favoring the occurrence of the overt disease.

The arterial function itself is very sensitive to the ageing process. Particularly at the level of large and medium-size arteries, the structure of the arterial walls progressively show increases in wall thickness due to collagen deposition, which in turn enhances the arterial stiffness and predisposes to endothelial dysfunction [2]. This process ultimately causes a series of secondary negative consequences on cardiac function and perfusion of peripheral organs and tissues, particularly at the level of the brain and the kidney microvasculature [3].

If this process is measured with the tape of the chronological age, the speed of such transition would be assumed to be the same in all individuals, no matter what kind of individual variability is present. However, in disagreement with this assumption, some individuals exhibit an accelerated ageing process (i.e., early vascular ageing), whereas others had a delayed age-associated arterial stiffening (super-normal vascular ageing), as it has been recently described [4].

Based on these premises, the view of arterial stiffness as a promising marker of vascular ageing might be well supported by the demonstration of its superiority over the chronological age in reflecting the process of vascular senescence. Indeed, measurement of arterial stiffness through pulse wave velocity (PWV) demonstrated ability in predicting the future occurrence of CV disease and death beyond chronological age and the presence of traditional CV risk factors. Finally, arterial stiffness as a measure of vascular ageing could be effective in quantifying how much of the normal process of vascular ageing is accelerated by the effect of concomitant CV risk factors on the arterial functions [5].

For a number of reasons, PWV measurement hasn't yet taken the stage of routine clinical practice. Indeed, PWV is time-consuming, in many cases it is operator-dependent and it requires dedicated equipment and trained operators. Recently, a method to estimate PWV (estimated PWV, ePWV) from age and blood pressure (mean arterial pressure, MAP) has been proposed [6] as an alternative way to obtain PWV values in each individual. Interestingly, ePWV was demonstrated to predict CV outcomes with the same accuracy as measured PWV (carotid-femoral PWV) and significantly better than traditional CV risk estimator equations, such as the SCORE system [7]. PWV estimation is based on the principle that age and mean arterial pressure (MAP), this latter conceived as a measure of distending pressure, are the two main determinants of arterial stiffness. Moreover, these two factors are integrated in polynomial equations which account for their possible interactions in determining PWV values. In other terms, the calculation of ePWV in each individual already accounts for the possible effects of ageing on blood pressure variations.

In the present issue of the *Journal*, Ji C et al. evaluated the prognostic ability of ePWV in predicting CV events and total mortality in a very large Chinese population (n = 98,348 individuals) enrolled in a community-based cohort study, the Kailuan Study [8]. Participants were evaluated over a period of about 10 years, during which the authors recorded a total of 6967 CV events and 9780 all-cause deaths. This impressive amount of data placed the authors in the best position to compare two different approaches for CV risk prediction, the former based on ePWV, the latter based on traditional CV risk estimation, which clearly have different background and rationale, but that are supposed to be affected by high co-linearity. The authors performed two different analyses. First, they tested the performance of ePWV in multivariate Cox proportional hazard regression models after adjustment for chronological age and a number of CV risk factors including blood pressure (specifically, pulse pressure). Furthermore, they tested the capacity of ePWV in effectively reclassifying the CV risk beyond the CV risk level estimated in any single individual using the China PAR score (which incorporates age and systolic BP), a CV risk prediction tool based on Chinese population. In the second analysis, they substantially repeated the first analysis but they removed chronological age from multivariate Cox regression models.

Results from the first analysis (which included chronological age among the list of independent determinants) showed that, for both outcomes, CV risk prediction based on ePWV (that is, on vascular age) was not significantly better than models based on chronological age. Indeed, adding the ePWV parameter to the model did not significantly improve its ability to reclassify the risk in any single individual. On the contrary, when chronological age was removed from the model, ePWV was found to significantly predict the occurrence of both CV events and all-cause death after adjustment for CV risk factors. Interestingly, in this second scenario, ePWV was found to be able in reclassifying CV risk at a more appropriate level than what proposed by the China PAR score, particularly in individuals with concomitant CV risk factors.

How should we interpret these results? Indeed, they tell us that CV risk estimation based on vascular age (assessed through ePWV) could be effective in improving risk classification beyond what it is usually done in clinical practice by applying traditional models based on chronological age for CV risk estimation. This reinforces the view that values of arterial stiffness effectively reflect the combined effect of ageing and CV risk factors on the arterial wall and, as a secondary consequence, on BP values.

However, on a closer view, the postulated superiority of models based on vascular age than on chronological age assumes more the features of a point victory rather than a striking knock-out. Indeed, as the authors correctly state, it is quite unusual to compare CV prediction models that do not account for the effect of chronological age as an independent variable. On the other side, since age and age squared are already included in equations for ePWV calculation, a double adjustment for these two parameters might have overweighed the overall influence of the age parameter on the models.

Other limitations should also be acknowledged. First, this approach is totally based on office BP, and do not account for inherent instability of this parameter. Indeed, the risk could be significantly underestimated after rapid BP decreases obtained by initiation of anti-hypertensive treatment. Second, MAP is calculated based on a fixed rule of thumb equation which was recently shown to be inaccurate in predicting true invasive MAP [9]. Third, the dichotomous representation of some CV risk factors which are well known to be continuously associated with CV risk, such as lipid levels, BP levels, BMI and glycated hemoglobin, often forces the variable to be flat within each category (presence/absence) with the result of an overall dilution of their associated prognostic significance.

In conclusions, results from the present study argue in favor of the clinical usefulness of arterial stiffness as a marker of vascular ageing. They also open the way to a broader use of mathematical models enabling the PWV estimation based on age and mean blood pressure, indicating that this measure could be included in CV risk estimation models, provided that these results will be confirmed in large prospective studies and different clinical contexts.

## Declaration of competing interest

None.
